# Interventional treatment of refractory non-traumatic chylous effusions in patients with lymphoproliferative disorders

**DOI:** 10.1007/s10238-024-01312-4

**Published:** 2024-03-30

**Authors:** Julia Wagenpfeil, Katharina Hoß, Andreas Henkel, Daniel Kütting, Julian Alexander Luetkens, Georg Feldmann, Peter Brossart, Ulrike Irmgard Attenberger, Claus Christian Pieper

**Affiliations:** 1 Division for Minimally-Invasive Lymph Vessel Therapy, Department of Diagnostic and Interventional Radiology, University Hospital Bonn, Venusberg-Campus 1, 53127 Bonn, Germany; 2 Center for Integrated Oncology Aachen Bonn Cologne Düsseldorf (CIO ABCD), Bonn, Germany; 3 Department of Internal Medicine III, University Hospital of Bonn, Bonn, Germany

**Keywords:** Lymphoproliferative disorders, Chylothorax, Chylous ascites, Lymphangiography, Embolization

## Abstract

To report results of interventional treatment of refractory non-traumatic abdomino-thoracic chylous effusions in patients with lymphoproliferative disorders. 17 patients (10 male; mean age 66.7 years) with lymphoproliferative disorders suffered from non-traumatic chylous effusions (chylothorax n = 11, chylous ascites n = 3, combined abdomino-thoracic effusion n = 3) refractory to chemotherapy and conservative therapy. All underwent x-ray lymphangiography with iodized-oil to evaluate for and at the same time treat lymphatic abnormalities (leakage, chylo-lymphatic reflux with/without obstruction of central drainage). In patients with identifiable active leakage additional lymph-vessel embolization was performed. Resolution of effusions was deemed as clinical success. Lymphangiography showed reflux in 8/17 (47%), leakage in 2/17 (11.8%), combined leakage and reflux in 3/17 (17.6%), lymphatic obstruction in 2/17 (11.8%) and normal findings in 2/17 cases (11.8%). 12/17 patients (70.6%) were treated by lymphangiography alone; 5/17 (29.4%) with leakage received additional embolization (all technically successful). Effusions resolved in 15/17 cases (88.2%); 10/12 (83.3%) resolved after lymphangiography alone and in 5/5 patients (100%) after embolization. Time-to-resolution of leakage was significantly shorter after embolization (within one day in all cases) than lymphangiography (median 9 [range 4–30] days; *p* = 0.001). There was no recurrence of symptoms or post-interventional complications during follow-up (median 445 [40–1555] days). Interventional-radiological treatment of refractory, non-traumatic lymphoma-induced chylous effusions is safe and effective. Lymphangiography identifies lymphatic abnormalities in the majority of patients and leads to resolution of effusions in > 80% of cases. Active leakage is found in only a third of patients and can be managed by additional embolization.

## Introduction

Chylous effusions (i.e. chylothorax, chylous ascites) can be caused by traumatic injury to the central lymphatic system, e.g., after surgical procedures, or can be due to non-traumatic causes with spontaneous occurrence of effusions (e.g., in malignancy or primary lymph vessel diseases) [1–5]. Non-traumatic chylous effusions are rare and occur in only about 2% of cases in patients with lymphoproliferative disorders [1, 3]. They are difficult to treat and can be fatal due to respiratory complications, immunosuppression, or nutritional wasting [1, 3, 6]. Furthermore, necessary oncologic treatment may be delayed by high-volume chylous discharge. In lymphoma patients, chylous effusions are thought to be caused by either direct infiltration of lymph vessels or by impeded lymphatic out-flow [3]. Therefore, treatment of the underlying lymphoproliferative disorder is imperative. Additionally, conservative measures such as parenteral nutrition or medium chain triglyceride (MCT) diet is applied in an effort to reduce lymph-flow in central lymphatics. When these treatment options fail, traditionally surgical treatment was attempted (e.g., ligation of the thoracic duct or pleurodesis). However this is often limited by patients’ general condition. More recently, interventional radiological treatment options are increasingly employed as less invasive alternatives [5]. X-ray lymphangiography with iodized-oil (XRL) is a theranostic procedure and can both identify the underlying lymphatic pathology and—at the same time—also have a therapeutic effect [7]. Dedicated lymphatic interventions (e.g., lymph vessel embolization) can additionally be useful for definitive leakage occlusion [5, 8].

However, so far only a small number of dedicated reports are available concerning efficacy of interventional treatment in lymphoma patients with spontaneous chylous effusions. The aim of this study is therefore to report results of interventional treatment of refractory non-traumatic abdomino-thoracic chylous effusions in patients with lymphoma.

## Material and methods

### Patient cohort

The local institutional review board approved retrospective data analysis with a waiver for additional informed patient consent (IRB approval number: 232/16). Medical records of consecutive patients suffering from lymphoproliferative and in one case with myeloproliferative disorders with concomitant non-traumatic chylous effusions undergoing a lymphatic intervention between 2018 and 2022 were reviewed.

The inclusion criteria were:

- Presence of clinically confirmed chylous effusions (triglycerides > 110mg/dl) [5] refractory to oncologic therapy and conservative treatment,
- XRL with or without additional lymph vessel embolization (LVE) performed at our institution,
- Available procedural, clinical and follow-up data.

Prior imaging and results of paracenteses were analyzed pre-interventionally to evaluate whether the patient was suffering from:

- Chylothorax/chylopericardium (thoracic effusions),
- Chylous ascites (abdominal effusions), or
- A combination thereof.

In total, 17 patients (10 male, 7 female; mean age 66.7 ± 13.5 [range 40–86] years) fulfilled the inclusion criteria (chylothorax n = 11, chylous ascites n = 3, combined abdomino-thoracic effusion n = 3) and were included into the study. Overall 8/14 patients (57%) with thoracic chylous effusions presented with bilateral, 5/14 (36%) with right-sided and 1/14 (7%) with left-sided chylothorax. Table 1 summarizes further patient characteristics.

**Table 1:** Patient characteristics

Parameter	Overall	Thoracic	Abdominal	Abdomino-thoracic
Number of patients (percentage)	17	11	3	3
Male: Female	10: 7	6: 5	2: 1	2: 1
Median age (range)	66.7 ± 13.5 years	70.2 ± 13.5 years	54.4 ± 15.8 years	66.5 ± 13.3 years
*Indication for lymphatic intervention*				
Thoracic (chylothorax)	11 (64.7%)			
Abdominal (chylous ascites)	3 (17.6%)			
Abdomino-thoracic (Combined chylothorax/chylous ascites)	3 (17.6%)			
*Etiology*				
Follicular lymphoma	6 (35.3%)	3	0	3
Diffuse large cell B-cell Lymphoma	4 (23.5%)	2	2	0
Chronic lymphocytic leukemia	3 (17.6%)	3	0	0
Mantle cell lymphoma	2 (11.8%)	2	0	0
M. Hodgkin	1 (5.9%)	0	1	0
Acute myeloid leukemia	1 (5.9%)	1	0	0
*Lymph node involvement*				
Mediastinal and retroperitoneal	13 (76.5%)	8	2	3
Only Mediastinal	4 (23.5%)	3	1	0
Comorbidities				
Cardiovascular disease	6 (35.3%)	3	2	1
Liver cirrhosis	1 (5.9%)	1	0	0
*Imaging findings*				
Chylo-lymphatic reflux	8 (47%)	4	2	2
Active lymphatic Leakage	2 (11.8%)	2	0	0
Combined lymphatic reflux and leakage	3 (17.6%)	3	0	0
Obstruction of central drainage	2 (11.8%)	1	0	1
Normal findings	2 (11.8%)	1	1	0

Characteristics of the entire patient cohort as well as patient subgroups with thoracic (chylothorax/chylopericardium), abdominal (chylous ascites) or abdomino-thoracic (chylothorax/chylopericardium and chylous ascites) effusions. *LAM* lymphangioleiomyomatosis

All patients had indwelling drainage catheters with daily drainage volumes ranging from 400 to 7000 ml (mean daily drainage volume 1812 ± 1930 ml). Cytology of the fluid showed no malignant cells within the effusions in any of the patients.

In all patients chylous effusions were refractory to dedicated oncological as well as conservative therapy. 10/17 patients previously received MCT-diet, 4/17 parenteral nutrition, and 3/17 both for 3–16 weeks (median 6 weeks). See Table 2 for detailed information on dedicated oncologic treatment.

**Table 2:** Treatment

Treatment	Overall	FL	DLBCL	CLL	MCL	MH	AML
*Stage*							
Binet B	3			3			
II	2	1	0		0	1	
III	6	3	1		2	0	
IV	5	2	3		0	0	
*Chemotherapy*							
R-CHOP	4	1	3	0	0	0	0
R-mini-CHOP	3	1	1	0	1	0	0
Ibrutinib	2	0	0	2	0	0	0
*Rituximab*							
Bendamustin	2	2	0	0	0	0	0
VXLD	1	0	0	0	1	0	0
Obinutuzumab	1	0	0	1	0	0	0
Rituximab	1	1	0	0	0	0	0
Rituximab, HD-MTX, Ifosfamid	1	1	0	0	0	0	0
BEACOPPesc	1	0	0	0	0	1	0
Stem cell transplantation	1	0	0	0	0	0	1
Radiation therapy	2	1	0	0	0	1	0
*Conservative treatment*							
MCT diet	10 (58.8%)	3	4	1	2	0	0
Parenteral nutrition	4 (23.5%)	2	0	1	0	1	0
both	3 (17.6%)	1	0	1	0	0	1

### Interventional technique

All examinations were performed by the same interventional radiologist (C.C.P., with 12 years of experience). Interventional techniques of nodal XRL and LVE have been described in detail before [4, 5, 9]. For nodal XRL, ultrasound-guided inguinal lymph node puncture was performed by using a 25-gauge needle in both groins with subsequent continuous slow application of iodized-oil (Lipiodol, Guerbet, France) by hand-injection under intermittent fluoroscopy. This was done to visualize the iliacal, retroperitoneal, and thoracic lymphatic system that drains lymph from the periphery and abdomen towards the lympho-venous junction.

**Fig. 1:**
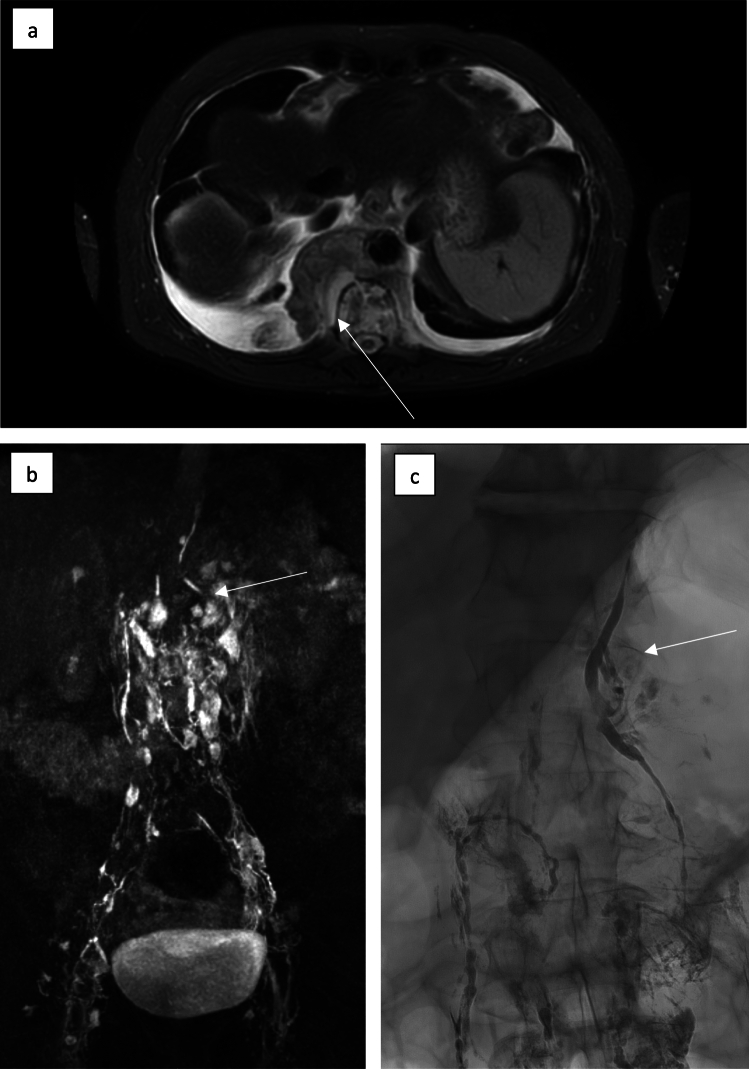
MR lymphangiography of a 56-year-old patient with extensive retrocural lymphoma manifestation (white arrow) (**a**). After nodal contrast application KM ascension via enhancement of pelvic and retroperitoneal lymphatic vessels is visible; in the upper abdomen the lower part of the thoracic duct can be seen. There is no further enhancement of the thoracic duct above the lymphoma mass corresponding to lymphatic obstruction (white arrow) (**b**). X-ray lymphangiography  corroborated  obstructive lymphatic drainage disorder at the level of the retrocrural lymphatic mass (white arrow) and consecutive chylolymphatic reflux in the upper abdomen (**c**)

**Fig. 2:**
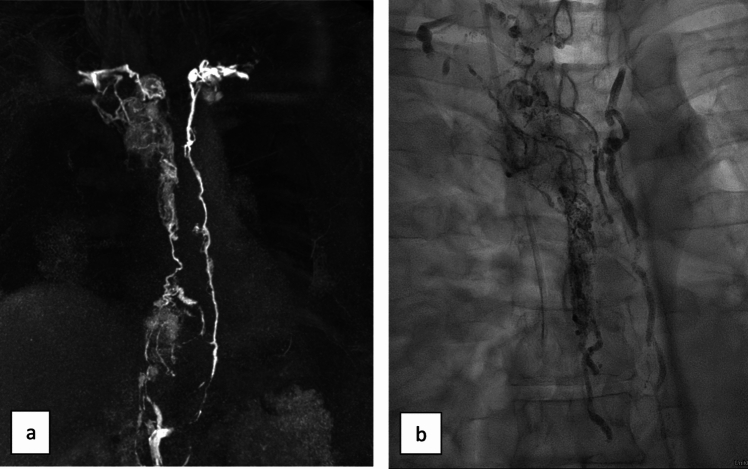
MR (**a**) and X-ray lymphangiography (**b**) of a 52-year-old patient with DLBCL and refractory non-traumatic chylothorax. There is a marked thoracic lymphatic flow disturbance with significant thoracic duct obstruction in the upper part and chylolymphatic reflux into alternative pathways in the middle and upper thirds of the thorax. Lymphatic enhancement was first visible in the alternative pathways on the right and only secondarily and delayed of the thoracic duct

Resulting X-ray lymphangiograms were evaluated intra-interventionally and categorized as follows:

- Chylo-lymphatic reflux (Figs. 1 and 2), i.e. lymph flow away from the central collecting and draining lymphatics (e.g. into mesenteric, pulmonary or pleural lymphatics),
- Active chylo-lymphatic leakage, i.e. pooling of contrast agent outside of discernable lymph vessels or nodes,
- Combined reflux and leakage,
- Obstruction of central lymphatic drainage (Fig. 1), i.e. impaired or occluded lymphatic outflow toward the venous junction or
- Normal findings, i.e. absence of any of the above stated abnormalities.

The further treatment procedure depended on the imaging findings: The intervention was stopped after XRL when reflux, obstruction or normal findings were seen. After that conservative treatment with MCT-diet was continued for at least 14 days. Primary LVE was performed in cases with identifiable active leakage. For this purpose, a transabdominal access to a larger retroperitoneal lymph vessel/the thoracic duct was established by fluoroscopic puncture using a ventral approach with a 22-gauge needle (e.g. Chiba, Cook Medical). After successful puncture, a micro-wire was introduced through this needle into the target lymphatic vessels [4] and the needle was then exchanged for a microcatheter. Further assessment of central lymphatic flow and anatomy was then done by injection of waters-soluble contrast agent via the microcatheter. The tip of the catheter was then placed as selectively as possible into the leaking lymph vessel and embolization was performed using a combination of coils (e.g.: Tornado, Cook Medical) and tissue adhesive (N-butyl cyanoacrylate, Histoacryl, Braun) mixed with iodized–oil or by tissue adhesive alone also as described in detail before [4, 6].

### Data acquisition and definitions

Clinical, procedural and follow-up data were collected retrospectively from the electronic patient files (Tables 1 and 2). XRL was defined as technically successful when enhancement of central lymphatics was observed; LVE when a target lymph vessel was successfully accessed and occluded by embolization. Clinical success was defined as resolution or significant reduction of lymphatic leakage without any further need for treatment. The Common Terminology Criteria for Adverse Events (CTCAE, Version 5) was used to categorize adverse events.

### Statistical analysis

Statistical analyses were performed employing SPSS, V27.0 (IBM, NY, USA). Descriptive statistics were done for patient characteristics and procedural parameters. Continuous variables are given as mean and standard deviation, discrete variables as count, median and range. The time-to-resolution of effusions was compared between XRL alone and LVE using the Mann–Whitney-U-test. Patient survival was analyzed using the Kaplan–Meier method. *P* values of < 0.05 were considered statistically significant.

## Results

### Imaging findings

Bilateral inguinal nodal XRL was performed using 20ml of iodized-oil and was technically successful in all patients. XRL showed chylo-lymphatic reflux in 8/17 (47%), active lymphatic leakage in 2/17 (11.8%), combined lymphatic reflux and leakage in 3/17 (17.6%), obstruction of central lymphatic flow in 2/17 (11.8%) and normal findings in 2/17 cases (11.8%) (Table 1).

In 2/3 patients with combined chlyo-lymphatic reflux and leakage, the leakage derived from refluxive vessels. In the remaining patient there was leakage immediately above the cisterna chyli and additional chylo-lymphatic reflux in the pelvis originating from the iliac lymphatics. Overall, the underlying cause of the effusion was identified in 15/17 cases (88.2%).

### Interventional treatment

According to the imaging-guided treatment strategy detailed above, 12/17 patients (70.6%) were treated by XRL alone, while 5/17 (29.4%) received additional LVE due to a detected active lymphatic leakage. 5/5 embolization procedures were technically successful. 4/5 of these patients suffered from chylothorax with leakage in the thorax and were treated by thoracic duct embolization (80%). 1/5 patients presented with combined chylothorax/chylous ascites with active leakage in the abdomen from retroperitoneal lymphatics. In this patient abdominal interstitial lymph node embolization was performed.

LVE was done with a combination of coils and tissue adhesive in 3/5 (60%) and with tissue adhesive alone in 2/5 patient (40%).

### Clinical course and complications

Overall, treatment was clinically successful in 15/17 (88.2%) cases. In 12/17 (70.6%) patients, chylous effusions resolved completely, in 3/17 (17.6%) cases effusions were reduced significantly so that no further drainage/treatment was necessary, while effusions persisted in 2/17 (11.8%) patients.

XRL alone was clinically successful in 10/12 (83.3%) (complete resolution 8/12; significant reduction 2/12). All embolization procedures (5/5) were clinically successful (complete resolution 4/5, significant reduction 1/5).

Both patients in whom treatment was unsuccessful suffered from follicular lymphoma. One patient with high-volume chylothorax (7000ml/day) showed normal findings on XRL without detectable leakage, reflux or lymphatic obstruction and therefore did not present with a viable target for LVE. The patient did subsequently not respond to treatment by XRL alone. He received pleurodesis two months later which led to resolution of chylous effusions. The other patient suffered from combined chylothorax and chylous ascites with extensive thoracic chylous reflux on XRL. Chylous effusions persisted after XRL, and the patient died 44 days later due to septicemia.

Time-to-resolution of effusions was significantly shorter after LVE (within one day in all cases) compared to XRL (median 9 days (range 4–30 days); *p* = 0.001).

Mean clinical follow-up time was 445 (40–1555) days. During follow-up there was no recurrence of treated effusions or development of new chylous effusions. No post-interventional complications were observed. At the end of the follow-up time 13/17 patients were alive. There was no 30-day mortality. Mean overall survival was 1154 days with a tendency towards shorter survival in patients without clinically successful treatment of chylous effusions (773 vs. 1193 days, *p* = 0.366).

## Discussion

Although refractory chylous effusions complicate lymphoma treatment in only 2% of cases, this accounts for over 10% of chylous effusions overall [10]. These effusions usually occur spontaneously without direct lymph vessel injury. Chylous effusions may be evident at the time of lymphoma diagnosis, but can also develop later during treatment [10].

As the cause of lymphatic leakage cannot be inferred from previous trauma or surgery, an exact work-up of lymphoma involvement and lymphatic imaging is of particular importance in non-traumatic effusions to elucidate the underlying lymphatic abnormality and plan targeted treatment [5]. Importantly, the course of a chylous effusion does not necessarily align with the success of treating the underlying lymphoma, and it can persist as a chronic issue despite successful oncologic treatment.

As reported previously [1, 11, 12], the majority of patients in our cohort suffered from follicular lymphoma or DLBCL. However, a wide range of different lymphoma types may lead to chylo-lymphatic effusions and it is currently not possible to predict the occurrence, location or course of effusions based on clinical parameters alone [11, 13]. The question remains which mechanism may lead to lymphatic effusions in lymphoma patients. In this respect three basic principles specifically in lymphoma-patients have been suggested [14–17]:

1. Lymph vessel (i.e. thoracic duct) rupture due to direct lymphoma infiltration leading to frank lymphatic leakage,
2. Obstruction of lymphatic run-off by compression of the thoracic duct by lymphoma-mass with subsequent backflow into pleural/peritoneal lymphatics,
3. High viscosity of the lymphatic fluid due to elevated numbers of cells/protein content leading to a pressure increase and therefore backflow.

Furthermore, interactions between the lymphatic system, the tumor characteristics as well as the body's reaction to the disease and treatment may also be additional factors in the development of chylous effusions not yet understood [15–17].

In both scenarios 2 and 3 distension of lymphatic vessels is thought to lead to an increased fragility of lymph-vessels with micro-ruptures already after minor trauma. However, the imaging findings in our cohort suggest that alterations in lymph-flow (possible due to lymph-node involvement and/or altered viscosity of lymph) with subsequent reflux into pleural/peritoneal lymphatics and seepage of lymph into the respective cavity represent the more prevalent mechanism of effusion formation in these patients (11/17 patients presented with reflux). This is corroborated by the fact that patients in the present cohort all had non-malignant effusions making direct infiltration of larger lymph-vessels unlikely. This is in line with a previously reported low rate of only 20% of lymphoma cells in chylous effusions [12, 18–22].

All patients in our cohort had mediastinal and/or retroperitoneal lymph node involvement on cross-sectional imaging. However, although all 3 patients with combined abdomino-thoracic effusions showed lymph-node enlargement both of mediastinal and retroperitoneal nodes, 8/11 patients showed mediastinal and retroperitoneal involvement, but presented with only a chylothorax. Additionally one patient only had enlarged lymph nodes in the mediastinum, but presented with chylous ascites. This underlined the importance of dedicated lymphatic imaging as chylo-lymphatic reflux and/or obstruction of lymphatic run-off rather than active localized leakage in the location of involved lymph-nodes could be identified as the cause of effusions in the majority of cases.

It has been proposed from single case studies that due to the anatomy of the thoracic duct, lymphoma-associated chylothorax generally occurs in the left hemithorax and that bilateral chylothorax is a rare condition [23–25]. Our data do not support this suggestion as left-sided chylothorax was seen only in 1/14 patients while 8/14 presented with bilateral and 5/14 with right-sided chylothorax. This is again most likely due to the fact that we observed chylo-lymphatic reflux rather than frank leakage from the thoracic duct to be the cause of the effusion in the majority of patients.

Owing to the rarity of the clinical problem, there is so far no standardized treatment strategy. Non-traumatic chylous effusions in general are more difficult to treat than the more common traumatic leakages [25–28]. Paradigmatic for DLBCL is the intricate interplay of factors such as lymphatic infiltration, angiogenesis, cell adhesion-mediated drug resistance (CAM-DR), tumor microenvironment and tumor progression that may contribute to the development of chylous effusions [12, 27, 29, 30].

Treatment usually involves drainage of the excess fluid and an initial conservative treatment attempt (dietary measures, octreotide/somatostatin), which aims at reducing lymphatic flow [4, 5, 9]. As chylo-lymphatic effusions may decrease in patients with lymphoma remission [11, 12], targeted treatment of the underlying disease is also important. However, especially in high-output effusions, chemotherapy alone may not be sufficient [3, 13]. In our cohort, all patients presented with effusions refractory to conservative and oncologic therapy. Interestingly, there is no definitive correlation between remaining lymphoma mass under successful treatment and resolution of effusions.

When conservative therapy fails, more invasive treatment options have to be considered. Traditionally surgical treatment was attempted by performing thoracic duct ligation or pleurodesis. However, identifying the thoracic duct, often occurring anatomical variations or alternate pathways during surgery can be problematic. Therefore “blind” ligation without knowledge of the exact cause and location of the underlying lymphatic pathology can be potentially dangerous and should be avoided. Pleurodesis can be effective in treating chylothorax [31], but is limited by several potentially long-lasting adverse events (e.g. pain, respiratory impairment) and often incomplete success especially in high-volume effusions [32]. Therefore, pleurodesis should nowadays be a bail-out procedure in patients not responding to less invasive alternatives.

In recent years, minimally-invasive procedures such as XRL or LVE have been shown to be safe and effective in treating chylous effusions [5]. However, only very few dedicated reports on interventional treatment of lymphoma-associated spontaneous chylous effusions are available with the largest group published so far including only 5 patients [1, 11].

XRL is can visualize the anatomy of and the flow within the central lymphatic system as well as associated pathologies [5]. This is particularly helpful for further treatment planning in non-traumatic chylous effusions as, in contrast to traumatic lymph vessel injuries, the underlying lymphatic abnormality is typically unknown prior to imaging. In addition, iodized-oil used for XRL can also have a therapeutic effect by sealing of leakage sites with considerably less morbidity compared to surgical approaches [1, 8, 11, 18, 33]. It is currently assumed that leaking iodized-oil directly blocks the leakage and that secondary sterile inflammation leads to formation of scar tissue [11, 34, 35]. In general, the therapeutic effect of XRL alone for thoracic lymphatic leakages varies considerably with success rates reported between 7 and 100% [4]. Higher success rates have been reported for traumatic lymphatic leakages (around 75%) while especially for non-traumatic chylous effusions clinical success rates seem to be considerably lower (around 20%) [36]. This is in line with a reported clinical success rate of only 20% for XRL also in patients with lymphoma-associated chylous effusions (n = 10) [37]. However, several factors such as the underlying disease, drainage volume as well as the amount of applied iodized-oil may also influence treatment success. In contrast to earlier reports we observed resolution of chylous effusions in > 80% of patients after XRL (with continued conservative treatment and chemotherapy). This higher rate of clinical success may be due to a higher dose of iodized-oil applied in our cohort. While Fukumoto and colleagues for example used only small amounts of contrast agent (6-12ml) in repeated interventions (up to 5 interventions per patient) [1], our patients all received XRL with 20 ml of iodized-oil in a single treatment session. This is in line with a recent report by Jardinet et al. [34] demonstrating a high success rate (83%) of high-volume iodized-oil XRL in traumatic chylothorax (mean dose per procedure, 75 ml). Of note, the amount applied by Jardinet et al. is several times higher than the manufacturer's recommended dose. At our institution we tend to limit the amount of applied iodized-oil to 20 ml in the average adult patient. Interestingly, we observed not only a higher clinical success rate of XRL, but resolution of the effusions also occurred earlier than reported previously. In addition to the effect of a higher dose of iodized-oil, the inclusion of patients with different lymphoma types or the volume of lymphatic output might also play a role in this respect [11, 13, 38].

It is so far impossible to predict when a therapeutic effect of XRL will set in with reported time intervals of days to several weeks [5]. The effect of additional direct LVE, in contrast, is more predictable and has a generally higher success rate [1]. If the thoracic duct can be intubated successfully, LVE is successful in well over 90% in patients with traumatic chylothorax. In contrast to XRL alone, LVE has recently be demonstrated to be effective in non-traumatic causes with a success rates of up to 85% [3, 37, 39–41]. This is in line with our results in patients with lymphoma-associated lymphatic leakage with clinical success in all patients undergoing LVE. Compared to XRL alone, LVE lead to a significantly faster resolution of the effusions (within only one days after embolization compared to a mean for 9 days after XRL). However, as obstruction of lymphatic out-flow seems to play a role in several of the patients with lymphoma-associated chylous effusions, we advocate to employ LVE sparingly and as selectively as possible in cases with identifiable active leakage or after failure of XRL alone. Further obstruction of lymphatic run-off by embolization (or ligation) may otherwise even worsen lymphatic leakage.

After successful treatment of the effusions, the positive effect of interventional treatment seems to be long lasting as we observed no recurrence of effusions within a mean clinical follow-up interval of over one year (up to 4 years). As reported before, interventional treatment is quite safe as post-interventional complications are rare and were not observed in our cohort. Considering high clinical success and low morbidity rates, interventional procedures should therefore be considered a primary treatment options in patients with refractory lymphoma-associated chylous effusions.

Our study has several limitations. First, data were analyzed retrospectively with inherent methodological limitations. Second, although—to our knowledge—being the largest patient cohort receiving interventional-radiological treatment of refractory lymphoma-associated chylous effusions, the sample size is still rather small due to the relatively rarity of this clinical problem. Third, the patient cohort was overall rather heterogeneous due to different underlying lymphoproliferative disorders. We therefore refrained from more in-depth statistical analysis. Since many patients were referred to us from outside hospitals for interventional treatment, data regarding the previous oncological therapies with regard to different chemotherapeutic agents are heterogeneous and only partially documented. In addition, the study was performed at a single center with all lymphangiographies being performed by the same interventionalist which may impair generalizability of the results. The source of the lymphatic effusions in patients with normal findings on imaging remains unclear to a certain extent. Lymphatic imaging was focused on the central lymphatic system at the time of treatment of the included patient cohort. Since then other imaging options such as mesenteric or hepatic lymphangiography have become available and might have shown abnormalities in the respective lymphatic systems as a source of the effusions [5]. Further research into long-term effects in larger, multi-center studies certainly is warranted.

In conclusion, interventional-radiological treatment of refractory, non-traumatic lymphoma-induced chylous effusions is safe and effective with a clinical success rate of 88%. Lymphangiography is helpful in identifying lymphatic abnormalities in the majority of patients and, at the same time, can already be therapeutic in > 80% of patients. Active lymphatic leakage is found in only a third of patients and can be managed by additional targeted lymph vessel embolization.

## Data Availability

The datasets generated and/or analyzed during the current study are available from the corresponding author on reasonable request.

## References

[1] Fukumoto A, Terao T, Kuzume A, Tabata R, Tsushima T, Miura D, Ikeda D, Kamura Y, Narita K, Takeuchi M, Matsue K. Management of lymphoma-associated chylothorax by interventional radiology and chemotherapy: a report of five cases. Int J Hematol. 2022;116:579–85. 10.1007/s12185-022-03397-7. 35819710 10.1007/s12185-022-03397-7

[2] Staats BA, Ellefson RD, Budahn LL, Dines DE, Prakash UB, Offord K. The lipoprotein profile of chylous and nonchylous pleural effusions. Mayo Clin Proc. 1980;55:700–4. 7442324

[3] Schild HH, Strassburg CP, Welz A, Kalff J. Treatment options in patients with chylothorax. Dtsch Arztebl Int. 2013;110:819–26. 10.3238/arztebl.2013.0819. 24333368 10.3238/arztebl.2013.0819 PMC3865492

[4] Pieper CC, Hur S, Sommer CM, Nadolski G, Maleux G, Kim J, Itkin M. Back to the future: lipiodol in lymphography-from diagnostics to theranostics. Invest Radiol. 2019;54:600–15. 10.1097/RLI.0000000000000578. 31283538 10.1097/RLI.0000000000000578

[5] Pieper CC. Back to the future II-a comprehensive update on the rapidly evolving field of lymphatic imaging and interventions. Invest Radiol. 2023;58:610–40. 10.1097/RLI.0000000000000966. 37058335 10.1097/RLI.0000000000000966

[6] Streitparth F, Theurich S, Streitparth T, Öcal O, Dos Santos DC, Flatz W. Treatment of refractory high-flow chylothorax in high-grade B-cell lymphoma by intratumoral lymphatic embolization. Cardiovasc Intervent Radiol. 2021;44:2002–4. 10.1007/s00270-021-02931-0. 34355251 10.1007/s00270-021-02931-0 PMC8626391

[7] Alejandre-Lafont E, Krompiec C, Rau WS, Krombach GA. Effectiveness of therapeutic lymphography on lymphatic leakage. Acta Radiol. 2011;52:305–11. 10.1258/ar.2010.090356. 21498367 10.1258/ar.2010.090356

[8] Schild HH, Naehle CP, Wilhelm KE, Kuhl CK, Thomas D, Meyer C, Textor J, Strunk H, Willinek WA, Pieper CC. Lymphatic interventions for treatment of chylothorax. Rofo. 2015;187:584–8. 10.1055/s-0034-1399438. 26090651 10.1055/s-0034-1399438

[9] Wagenpfeil J, Kupczyk PA, Henkel A, Geiger S, Köster T, Luetkens JA, Schild HH, Attenberger UI, Pieper CC. Ultrasound-guided needle positioning for nodal dynamic contrast-enhanced MR lymphangiography. Sci Rep. 2022;12:3621. 10.1038/s41598-022-07359-1. 35256625 10.1038/s41598-022-07359-1 PMC8901837

[10] Doerr CH, Allen MS, Nichols FC 3rd, Ryu JH. Etiology of chylothorax in 203 patients. Mayo Clin Proc. 2005;80:867–70. 10.4065/80.7.867. 16007891 10.4065/80.7.867

[11] Pospiskova J, Smolej L, Belada D, Simkovic M, Motyckova M, Sykorova A, Stepankova P, Zak P. Experiences in the treatment of refractory chylothorax associated with lymphoproliferative disorders. Orphanet J Rare Dis. 2019;14:9. 10.1186/s13023-018-0991-3. 30626415 10.1186/s13023-018-0991-3 PMC6327395

[12] Teng CL, Li KW, Yu JT, Hsu SL, Wang RC, Hwang WL. Malignancy-associated chylothorax: a 20-year study of 18 patients from a single institution. Eur J Cancer Care (Engl). 2012;21:599–605. 10.1111/j.1365-2354.2012.01329.x. 22309398 10.1111/j.1365-2354.2012.01329.x

[13] O’Callaghan AM, Mead GM. Chylothorax in lymphoma: mechanisms and management. Ann Oncol. 1995;6:603–7. 10.1093/oxfordjournals.annonc.a059251. 8573541 10.1093/oxfordjournals.annonc.a059251

[14] Talwar A, Lee HJ. A contemporary review of chylothorax. Indian J Chest Dis Allied Sci. 2008;50:343–51. 19035053

[15] Laba JM, Nguyen TK, Boldt RG, Louie AV. Spontaneous resolution of chylothorax-associated lymphoma treated with external beam radiotherapy: a case report and comprehensive review of the literature. Cureus. 2016. 10.7759/cureus.761. 27733965 10.7759/cureus.761 PMC5045324

[16] Allen CJ, DiPasco PJ, Koshenkov V, Franceschi D. Non-Hodgkin’s lymphoma as a risk factor for persistent chylothorax after transhiatal esophagectomy. World J Oncol. 2012;3:233–5. 10.4021/wjon523w. 29147312 10.4021/wjon523w PMC5649902

[17] Maranatha D, Bestari B. Problema diagnostik dan respons kemoterapi pada seorang penderita classical limfoma hodgkin tipe mixed cellularity dengan temporary spontaneus regression: [Difficult case of classical hodgkin lymphoma mixed cellularity type with temporary spontaneous Regression]. J Respirasi. 2017;3:7–11.

[18] Ekeke CN, Chan EG, Luketich JD, Dhupar R. Delayed chylothorax during treatment of follicular lymphoma with a malignant pleural effusion. Case Rep Surg. 2020. 10.1155/2020/2893942. 32158584 10.1155/2020/2893942 PMC7061108

[19] Van De Voorde L, Vanneste B, Borger J, Troost EGC, Werner P. Rapid decline of follicular lymphoma-associated chylothorax after low dose radiotherapy to retroperitoneal lymphoma localization. Case Rep Hematol. 2014. 10.1155/2014/684689. 24891961 10.1155/2014/684689 PMC4033525

[20] Paul T, Yadav DK, Alhamar M, Dabak V. Primary pleural extranodal marginal zone lymphoma presenting as bilateral chylothorax. Case Rep Oncol. 2020;13:929–34. 10.1159/000508704. 32884542 10.1159/000508704 PMC7443639

[21] Sammartino D, Khanijo S, Koenig S, Katsetos JF, Tufano A, Rai KR, et al. Chylothorax in patients with chronic lymphocytic leukemia: a case series. J Hematol. 2018;7:14–8. 10.14740/jh339w. 30294401 10.14740/jh339w PMC6173329

[22] Kohmoto O, Kawabe K, Ono H, Yanagimoto R, Arimoto J, Hatada A, et al. Chylothorax associated with chronic lymphocytic leukemia. Intern Med. 2016;55:3641–4. 10.2169/internalmedicine.55.7250. 27980266 10.2169/internalmedicine.55.7250 PMC5283966

[23] Barillas S, Rodas A, Ardebol J, Martí JL. Nontraumatic chylothorax secondary to lymphoma and filariasis. J Surg Case Rep. 2020. 10.1093/jscr/rjaa309. 32983405 10.1093/jscr/rjaa309 PMC7502309

[24] Janjetovic S, Janning M, Daukeva L, Bokemeyer C, Fiedler W. Chylothorax in a patient with Hodgkin’s lymphoma: a case report and review of the literature. Tumori. 2013. 10.1177/030089161309900324. 24158090 10.1177/030089161309900324

[25] Wijaya SY, Koesoemoprodjo W. Indonesian female with bilateral chylothorax and mediastinal non-Hodgkin lymphoma: a case report. Int J Surg Case Rep. 2022;102: 107827. 10.1016/j.ijscr.2022.107827. 36473268 10.1016/j.ijscr.2022.107827 PMC9723926

[26] Nadolski G. Nontraumatic chylothorax: diagnostic algorithm and treatment options. Tech Vasc Interv Radiol. 2016;19:286–90. 10.1053/j.tvir.2016.10.008. 27993324 10.1053/j.tvir.2016.10.008

[27] Goity LD, Itkin M, Nadolski G. An algorithmic approach to minimally invasive management of nontraumatic chylothorax. Semin Intervent Radiol. 2020;37:269–73. 10.1055/s-0040-1713444. 32773952 10.1055/s-0040-1713444 PMC7394565

[28] Ur Rehman K, Sivakumar P. Non-traumatic chylothorax: diagnostic and therapeutic strategies. Breathe (Sheff). 2022;18: 210163. 10.1183/20734735.0163-2021. 36337134 10.1183/20734735.0163-2021 PMC9584559

[29] Fantin A, Castaldo N, Vailati P, Morana G, Orso D, Vetrugno L, Patruno V. Pleural effusion aetiology, presentation, treatment and outcome in haematological diseases: a review. Acta Biomed. 2021;92: e2021268. 10.23750/abm.v92i5.11794. 34738567 10.23750/abm.v92i5.11794 PMC8689299

[30] Skouras V, Kalomenidis I. Chylothorax: diagnostic approach. Curr Opin Pulm. 2010;16:387–93. 10.1097/MCP.0b013e328338dde2. 10.1097/MCP.0b013e328338dde2 20410823

[31] Mares DC, Mathur PN. Medical thoracoscopic talc pleurodesis for chylothorax due to lymphoma: a case series. Chest. 1998;114:731–5. 10.1378/chest.114.3.731. 9743158 10.1378/chest.114.3.731

[32] Shaw P, Agarwal R. Pleurodesis for malignant pleural effusions. Cochrane Database Syst Rev. 2004. 10.1002/14651858.CD002916.pub2. 14973997 10.1002/14651858.CD002916.pub2

[33] Kos S, Haueisen H, Lachmund U, Roeren T. Lymphangiography: forgotten tool or rising star in the diagnosis and therapy of postoperative lymphatic vessel leakage. Cardiovasc Intervent Radiol. 2007;30:968–73. 10.1007/s00270-007-9026-5. 17508245 10.1007/s00270-007-9026-5

[34] Jardinet T, Veer HV, Nafteux P, Depypere L, Coosemans W, Maleux G. Intranodal lymphangiography with high-dose ethiodized oil shows efficient results in patients with refractory, high-output postsurgical chylothorax: a retrospective study. AJR Am J Roentgenol. 2021;217:433–8. 10.2214/AJR.20.23465. 34106766 10.2214/AJR.20.23465

[35] Youssef EW, Aly A, Brahmbhatt A, Moussa A, Santos E. Lymphatic interventions in the cancer patient. Curr Oncol Rep. 2022;24:1351–61. 10.1007/s11912-022-01293-1. 35639331 10.1007/s11912-022-01293-1

[36] Kaminski LC, Wagenpfeil J, Buermann J, Lutz PL, Luetkens JA, Attenberger UI, Strassburg CP, Kalff JC, Schild HH, Pieper CC. Long-term clinical outcome of abdomino-thoracic lymphatic interventions of traumatic and non-traumatic lymphatic leakage in adults. Biomedicines. 2023;11:2556. 10.3390/biomedicines11092556. 37760997 10.3390/biomedicines11092556 PMC10526188

[37] Nadolski GJ, Itkin M. Thoracic duct embolization for nontraumatic chylous effusion: experience in 34 patients. Chest. 2013;143:158–63. 10.1378/chest.12-0526. 22797603 10.1378/chest.12-0526

[38] Yannes M, Shin D, McCluskey K, Varma R, Santos E. Comparative analysis of intranodal lymphangiography with percutaneous intervention for post-surgical chylous effusions. J Vasc Interv Radiol. 2017;28:704–11. 10.1016/j.jvir.2016.12.1209. 28169139 10.1016/j.jvir.2016.12.1209

[39] Kim PH, Tsauo J, Shin JH. Lymphatic interventions for chylothorax: a systematic review and meta-analysis. J Vasc Interv Radiol. 2018;29:194–202. 10.1016/j.jvir.2017.10.006. 29287962 10.1016/j.jvir.2017.10.006

[40] Johnson OW, Chick JF, Chauhan NR, Fairchild AH, Fan CM, Stecker MS, Killoran TP, Suzuki-Han A. The thoracic duct: clinical importance, anatomic variation, imaging, and embolization. Eur Radiol. 2016;26:2482–93. 10.1007/s00330-015-4112-6. 26628065 10.1007/s00330-015-4112-6

[41] Chen E, Itkin M. Thoracic duct embolization for chylous leaks. Semin Intervent Radiol. 2011;28:63–74. 10.1055/s-0031-1273941. 22379277 10.1055/s-0031-1273941 PMC3140251

